# Cysteine protects rabbit spermatozoa against reactive oxygen species-induced damages

**DOI:** 10.1371/journal.pone.0181110

**Published:** 2017-07-10

**Authors:** Zhendong Zhu, Zhanjun Ren, Xiaoteng Fan, Yang Pan, Shan Lv, Chuanying Pan, Anmin Lei, Wenxian Zeng

**Affiliations:** 1 College of Animal Science and Technology, Northwest A&F University, Yangling, Shaanxi, China; 2 College of Veterinary Medicine, Northwest A&F University, Yangling, Shaanxi, China; Universite Blaise Pascal, FRANCE

## Abstract

The process of cryopreservation results in over-production of reactive oxygen species, which is extremely detrimental to spermatozoa. The aim of this study was to investigate whether addition of cysteine to freezing extender would facilitate the cryosurvival of rabbit spermatozoa, and if so, how cysteine protects spermatozoa from cryodamages. Freshly ejaculated semen was diluted with Tris-citrate-glucose extender supplemented with different concentrations of cysteine. The motility, intact acrosomes, membrane integrity, mitochondrial potentials, 8-hydroxyguanosine level and sperm-zona pellucida binding capacity were examined. Furthermore, glutathione peroxidase (GPx) activity, glutathione content (GSH), and level of reactive oxygen species (ROS) and hydrogen peroxide of spermatozoa were analyzed. The values of motility, intact acrosomes, membrane integrity, mitochondrial potentials and sperm-zona pellucida binding capacity of the frozen-thawed spermatozoa in the treatment of cysteine were significantly higher than those of the control. Addition of cysteine to extenders improved the GPx activity and GSH content of spermatozoa, while lowered the ROS, DNA oxidative alterations and lipid peroxidation level, which makes spermatozoa avoid ROS to attack DNA, the plasma membrane and mitochondria. In conclusion, cysteine protects spermatozoa against ROS-induced damages during cryopreservation and post-thaw incubation. Addition of cysteine is recommended to facilitate the improvement of semen preservation for the rabbit breeding industry.

## Introduction

Cryopreservation of spermatozoa has long been an important strategy for preservation of male fertility. However, the quality of the frozen-thawed spermatozoa is extremely affected during preservation [1]. Spermatozoa contain large amounts of unsaturated fatty acids in the plasma membrane, making them highly susceptible to reactive oxygen species (ROS) stress [2]. Unfortunately, the process of cryopreservation lead to accumulation of ROS [3]. ROS may cause lipid peroxidation (LPO) in cell membranes [4], which is associated with damage such as extensive structural alterations, irreversible loss of motility, a profound change in metabolism, and a high rate of leakage of intracellular cell constituents [5]. Therefore, addition of antioxidants to freezing extender may prevent spermatozoa from oxidative stress.

Cysteine, one component of glutathione, contains thiol groups and may penetrate the plasma membrane easily [6]. Uysal and Bucak (2007) [7] reported that cysteine enhanced intracellular glutathione (GSH) biosynthesis and protected the proteins, DNA and membrane lipids because of the direct radical-scavenging ability of GSH. It has been shown that cysteine protects spermatozoa against cryo-damage in boars [8, 9], bulls [10, 11], buffalos [12–14], dogs [15], chickens [16], goats [17–19], cats [20], and rams [6, 7, 21, 22]. However, the underline mechanisms are still unclear. Therefore, the aims of this study were elucidate whether cysteine improves sperm tolerance through its anti-oxidative activity and determine whether addition of cysteine protects spermatozoa from ROS stress at each step during the preservation.

## Materials and methods

### Chemicals

All chemicals were purchased from Sigma (Shanghai, China), unless specified.

### Extender preparation

The basic extender was a Tris-citrate-glucose extender (TCG), composed of 250 mM Tris-hydroxymethylaminomethane, 87.5 mM citric acid, 69 mM glucose, 100 million IU penicillin sodium and 100 million IU streptomycin sulphate, pH 6.8 [23]. The capacitation medium [24] was TCG with 5 mM CaCl_2_, 25 mM NaHCO_3_, 7 mg/mL BSA, 10 μg/mL heparin. The freezing extenders [25] were TCG supplemented with 20% (v/v) egg yolk, 4% (v/v) DMSO (final concentration) and L-cysteine. The final concentrations of L-cysteine in TCG extender were 0, 2.5, 5, 7.5,10 mM.

### Animals

All experimental procedures involving animals were approved by the Northwest A&F University’s Institutional Animal Care and Use Committee (Approval ID: 2011ZX08008-002). Fifteen 1-year-old male rabbits (body weights 3.0–4.5 kg) were used in this study. Rabbit were kept in single cages with a controlled photoperiod of 16 h of light and 8 h of darkness, fed with a commercial standard diet, and allowed free access to water. Two mature female rabbits were used as teasers for collection of semen.

### Semen processing

Semen was collected by using an artificial vagina on a regular basis (two collections per week). Ejaculates (n = 15) were pooled to avoid individual animal differences. The semen was placed in warm water (25°C) and delivered to the laboratory within an hour for evaluation. Semen samples with over 90% motile spermatozoa were pooled.

The sperm concentrations were determined by haemocytometry. The freshly collected semen was diluted with TCG and gradually cooled to 5°C. The semen was diluted with the same volume of freezing extenders and kept for 30 min at 5°C. The diluted semen was packed into 0.25 mL-straws immediately (3×10^7^ cells/ straw). The straws were placed horizontally 5 cm above the surface of liquid nitrogen for 10 min, and then plunged into it. After storage in liquid nitrogen for at least 7 d, the frozen semen was thawed in a water bath at 37°C for 30 sec.

At least 10 straws for each group were frozen from each collection of semen. Each time, two straws from each group were thawed and pooled for assessing sperm parameters. The experiments were performed a triplicates.

### Sperm motility

Two straws of frozen semen from each experiment group were transferred into a water bath at 37°C for 30 sec. The thawed-semen was pooled together as one sample, diluted with TCG extender (1:4) and incubated in a water bath at 37°C for 15 min. Sperm motility was evaluated by visual estimation. A 10-μL drop of sperm suspension was delivered onto a pre-warmed clean glass slide, and covered with a clean coverslip. The slides were examined in an optical microscope with a bright field lens (Nikon 80i; Tokyo, Japan) at 200X total magnification. The rate of motile sperm was referred to as the sperm motility rate. Sperm motility was estimated after viewing five different fields.

### Acrosomal intactness

For the acrosome staining, a protocol described by Zhu et al. (2015) [23] was used after a slight modification. Briefly, 30 μL of the sperm samples was smeared onto a clean glass slide, air-dried, then fixed with absolute methanol for 10 min. Then, 30 μL fluorescein isothiocyanate-peanut agglutinin (FITC-PNA, Sigma) solution (100 μg/mL) diluted in PBS was spread over each slide. The slides were incubated in a dark and moist chamber at 37°C for 30 min, and subsequently washed with PBS twice and air-dried in the dark. Ten microliters of antifade solution (P0123; Beyotime Institute of Biotechnology, Shanghai, China) was added to the slide to preserve fluorescence before a clean coverslip was applied. The edges of the coverslip were sealed with colorless nail polish.

**Fig 1:**
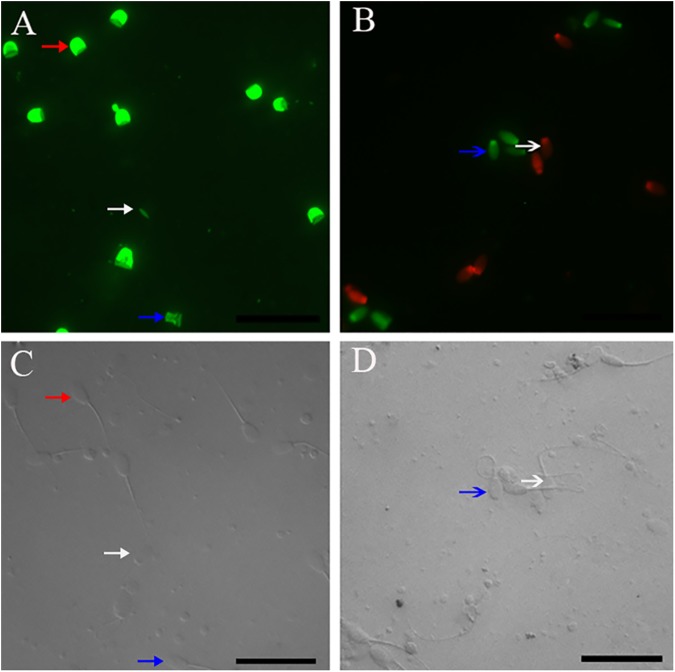
Photomicrographs of the post-thaw rabbit spermatozoa. Images(C, D) obtained under phase contrast microscopy. (A) Image obtained by FITC-labeled peanut agglutinin staining, Images (A) and (C) are from the same field. Red arrow indicates intact acrosomes, spermatozoa with intensively bright fluorescence of the acrosomal cap which were indicated by an intact outer acrosomal membrane; white arrow indicates damaged acrosome, spermatozoa with no fluorescence which were indicated by a complete loss of the outer acrosomal membrane and was determined under a phase contrast illumination system; blue arrow indicates partially damaged acrosome, spermatozoa with disrupted fluorescence of the acrosomal cap which were indicated by partial disruption of the acrosomal membrane; (B) Image obtained by SYBR-14 plus propidium ioide (PI) staining. Images (B) and (D) were from the same field. Blue arrow indicates membrane integrity, which were stained green with SYBR-14 but not with PI; white arrow indicates membrane damaged, which were stained red with PI but not with SYBR-14. Scale bars represent 30 μm.

The acrosomal status of the spermatozoa was monitored and photographed in an epifluoresence microscope (Nikon 80i; Tokyo, Japan) with a set of filters (400X) with 488 nm excitation and 525 nm emission. As shown in Fig 1A, the observed fluorescence images of spermatozoa stained with FITC-PNA were classified into three groups: intact acrosome, partially damaged acrosome (including intermediate form of acrosome reactivity and partially cryo-damaged), and damaged acrosome (including acrosome reacted and acrosome cryo-damaged). For the same field, photographs were also taken with a phase-contrast microscope. At least 200 spermatozoa per slide were assessed. Three separate aliquots (replicates) were assessed from each semen sample. All samples were identified and evaluated by one observer (ZZD).

### Sperm membrane integrity

SYBR-14 and propidium iodide (Sperm Viability Kit Molecular Probes, Leiden, The Netherlands, L7011) were used to evaluate membrane integrity [23]. Briefly, aliquots of 20 μL sperm supernatant were added to 100 μL HEPES-buffered saline solution (10 mM HEPES, 150 mM NaCl, 10% (w/v) BSA, pH 7.4), and stained with 0.12 μL SYBR-14 working solution (100 μM in DMSO) for 10 min at 36°C in the dark, followed by addition of 0.6 μL propidium iodide (PI) stock solution (2.4 mM in water), then incubated for additional 10 min.

The staining was monitored and photographed in an epifluoresence microscope (Nikon 80i; Tokyo, Japan) with a set of filters (400X) with 535 nm excitation and 617 nm emission for PI red fluorescence and 488 nm excitation and 516 nm emission for SYBR-14 green fluorescence. The spermatozoa were classified into two groups: Group A showing membrane integrity, Group B showing membrane damage (Fig 1B). At least 200 spermatozoa per slide were assessed. Three separate aliquots (replicates) were assessed from each semen sample. All samples were identified and evaluated by one observer (ZZD).

### Mitochondrial membrane potentials (ψm)

The changes of sperm mitochondrial membrane potential (ΔΨm) were evaluated using a JC-1 (lipophilic cation 5,5^’^,6,6’-tetrachloro-1,1’,3,3’ -tetraethylbenzimidazolcarbocyanine iodide) Mitochondrial Membrane Potential Detection Kit (Beyotime Institute of Biotechnology), by following the manufacturer’s instruction [23]. Briefly, the sperm samples (2×10^6^/mL) were stained with 28 μL of JC-1 (stock solution) in PBS (final volume, 100 μL). After being incubated at 37°C for 30 min in the dark, the samples were centrifuged at 600 × g for 5 min, and re-suspended in JC-1 buffer and placed on ice. Sperm samples were immediately analyzed in a flow cytometer (FAC-SCalibur, BD Biosciences) with excitation at 525 nm and emission at 590 nm. A total of 20 000 sperm-specific events were analyzed and the percentage of spermatozoa with red fluorescence in total sperm number was calculated. Data were processed by using the CellQuest program (BD Biosciences).

### Lipid peroxidation

As in previous reports [23], BODIPY 581/591C_11_ (Molecular Probes), a sensitive fluorescent probe for lipid peroxidation (LPO), was used to measure LPO. Briefly, sperm samples were stained with the BODIPY 581/591C_11_ probe (working solution: 10 μΜ), and incubated at 37°C for 30 min in the dark, then washed with PBS to remove the unbound probe, and analyzed with a flow cytometer (FACSCalibur, BD Biosciences). Red fluorescence was measured using an FL2 longpass filter (>670 nm). A total of 20,000 sperm-specific events were evaluated and calculated as percentages. Data were processed by using the CellQuest program (BD Biosciences).

### Detection of 8-hydroxyguanosine (8-OHdG) by immunofluorescence

The generation of the oxidized base adduct, 8-OHdG was detected as a biomarker for oxidative DNA damage [26]. Sperm samples were washed twice in PBS and subsequently diluted to a final concentration of 5×10^6^ sperm/mL. Spermatozoa were fixed using 4% paraformaldehyde in PBS for 20 min at room temperature. Fixed sperm were washed three times with PBS, samples were resuspended in permeabilization solution (0.25% Triton X-100) for 10 minutes. The sample subsequently washed twice with PBS after permeabilization, then treated for 1 h at 37°C with 100 μL of blocking solution (20% goat serum) in PBS for blocking the nonspecific binding sites. Sperm samples were incubated (1h at 37 ^o^C) in 50 μL 0.1% Triton X-100 containing a mouse monoclonal antibody to 8-OHdG (Santa Cruz Biotechnology), diluted (1:50) in a PBS solution with 2% BSA. After washing twice, spermatozoa were incubated in the darkness (1 h at 37 ^o^C) with a FITC-conjugated goat anti-mouse IgG antibody (Santa Cruz Biotechnology) diluted 1: 100 in PBS. Then, samples were washed twice, resuspended in 500 μL PBS for flow cytometric analysis. FL1 (green fluorescence, dichroic long pass 550 nm, bandpass filter 525 nm, detection width 505–545 nm) was used to detect the 8-OHdG level of post-thaw spermatozoa. A total of 20,000 sperm-specific events were evaluated and calculated as percentages. Data were processed by using the CellQuest program (BD Biosciences). The samples were also viewed and photographed using an epifluorescence microscope (80i; Nikon) with a set of filters (400×). A negative control with mouse IgG instead of the anti-8-OHdG antibody and a positive control treated with 2 mM H_2_O_2_ and 1 mM FeCl_2▪_4H_2_O were included to ensure assay specificity.

### Sperm-zona pellucida (ZP) binding capacity

The porcine ovaries were collected from an abattoir. As described by previous studies [27–29], The cumulus-oocyte complexes were isolated and incubated in M199 (Life Technologies Corporation, Beijing, China) supplemented with 3 mg/mL hyaluronidase at 38.5°C for 5 min, followed by gentle pipetting for 3 to 5 min to remove cumulus cells. The zona pellucidas were prepared from the cumulus cell-free oocytes using a microinjector. Twenty-five zona pellucidas were placed in a 50 μL droplet of TCG medium for each group. Meanwhile, the frozen spermatozoa were thawed and incubated with capacitation medium for 3 h. Then 50 μL of the capacitated sperm suspension were added into the TCG droplet containing 25 zona pellucidas, and incubated for 2 h at 39^°^C in a humidified atmosphere saturated with 5% CO2. Following incubation, the sperm-zona pellucida complexes were gently rinsed three times with PBS using a bore pipette to remove the loosely attached spermatozoa. The sperm tightly bound to each of zona pellucida was also removed by repeated aspiration using a narrow-bore pipette, and were counted by one observer (ZZD) under a microscope. The total number of sperm bound to zona pellucida were counted.

### Measurement of intracellular reactive oxygen species

The Reactive Oxygen Species Assay Kit (Beyotime Institute of Biotechnology) was applied to measure the level of intracellular ROS by following the manufacturer’s instruction. 2′,7′-dichlorodihydrofluorescein diacetate (DCFH-DA) is oxidized by reactive oxygen species to 2′, 7′-dichlorofluorescein (DCF) which is highly fluorescent at 530 nm. Briefly, the sperm pellet were suspended with TCG extender containing 10 μM DCFH-DA (10×10^6^ cells /mL), and incubated for 30 min at 37°C in the dark. The relative levels of fluorescence were quantified by a multi-detection microplate reader (485 nm excitation and 535 nm emission (Synergy HT, BioTek, Vermont, USA).

### Measurement of intracellular hydrogen peroxide

The concentration of intracellular hydrogen peroxide (H_2_O_2_**)** in spermatozoa was measured with a Hydrogen Peroxide Assay Kit (Beyotime Institute of Biotechnology), following the manufacturer’s instruction. The assay is based on formation of a violet complex between xylenol orange and ferric iron, which is produced by the hydrogen peroxide. The colorimetric reaction could be detected by microplate reader at a wavelength of 560 nm. Briefly, 1×10^6^ spermatozoa were re-suspended with 100 μL of lysis solution. The samples were ultrasonically lysed (20KHz, 750W, operating at 40%, on 3s, off 5s, 5 cycles) on ice and centrifuged at 12 000×g for 5 min. Subsequently, 100 μL of the supernatants were mixed with 100 μL of test solutions in the 96-well plates, incubated at room temperature for 20 min and measured immediately with a microplate reader (Synergy HT) at a wavelength of 560 nm. The concentration of H_2_O_2_ released was calculated according to standard concentration curve originating from standard solutions. The concentrations of H_2_O_2_ in the standard curve were 1, 2, 5, 10, 20, 50 and 100 μM. The analyses were performed in triplicate.

### Determination of glutathione content in spermatozoa

Glutathione (GSH) levels were determined by using a Glutathione Quantification Kit (Beyotime Institute of Biotechnology). GSH level was determined by measuring absorption at 412 nm for the reaction of DTNB (5,5’-dithiobis (2-nitrobenzoicacid)) with GSH that generates a yellow product, 2-nitro-5-thiobenzoic acid [30]. Sperm samples were centrifuged at 1000 ×g for 5 min at 25 ^o^C. The pellets were re-suspended in three parts of TCG, and lysed by three cycles of rapid cooling in liquid nitrogen and thawing at 37.8°C, centrifuged at 10 000 ×g for 10 min. The supernatants were transferred to a 96-well plate to detect the concentration of GSH in spermatozoa following the manufacturer’s instructions. The concentration of GSH was expressed as nM per10^8^ spermatozoa. The analyses were performed in triplicate.

### Analysis of glutathione peroxidase activity

Glutathione peroxidase (GPx) activity were measured with a Total Glutathione Peroxidase Assay kit (Beyotime Institute of Biotechnology). GPx activity was assayed by quantifying the rate of oxidation of the reduced glutathione to the oxidized glutathione by cumene hydroperoxide (Cum-OOH) catalyzed by GPx, and the GPx activity was calculated by measuring the reduction of NADPH to NADP^+^ at 340 nm of absorbance. Briefly, 1×10^6^ spermatozoa were re-suspended in 100 μL of lysis solution, lysed ultrasonically (20KHz, 750W, operating at 40%, on 3s, off 5s, 5 cycles) on ice and centrifuged at 12 000 ×g for 10 min at 4°C. The supernatants were added to a 96-well plate for analysis of glutathione peroxidase activity in a microplate reader at 340 nm following the manufacturer`s instructions. The analyses were performed in triplicate.

### Statistical analysis

All data were first tested for normality and variance homogeneity through the Shapiro–Wilk and Levene tests respectively. When necessary, data were transformed by arc-sin square root transformation prior to statistical analysis. Data were analyzed by a general mixed model (with repeated measures), followed by multiple comparisons with the Tukey test by using SPSS version 17.0 for Windows (SPSS Inc., Chicago, IL, USA). In this model, the treatment was the inter-subject factor, and cooling, equilibrium and incubation time (0 h, 1 h, 2 h) were the intrasubject factor. In all cases, each functional parameter was the dependent variable. All the values are presented as mean ± standard error of the mean (SEM). Treatments were considered statistically different from one another at p < 0.05.

## Experimental design

**Fig 2:**
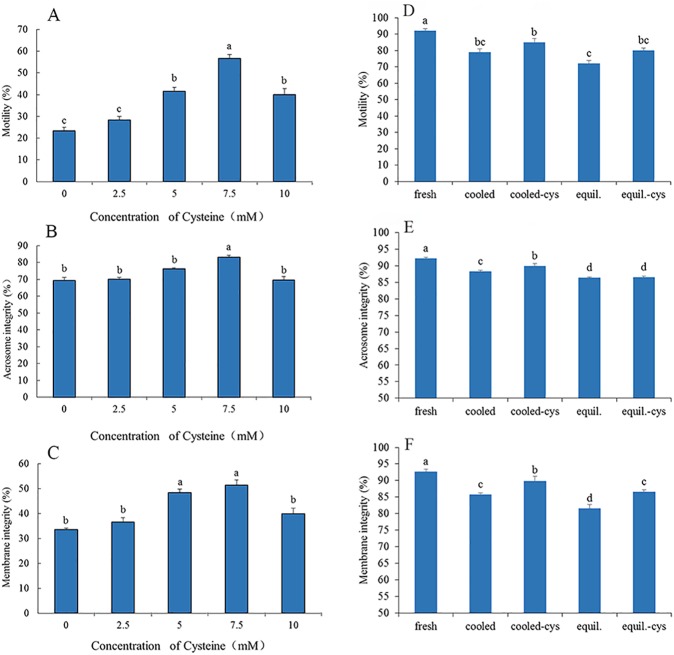
Spermatozoa parameters of the frozen-thawed rabbit spermatozoa supplementation of different concentrations of cysteine (A-C) And spermatozoa exposure with 7.5 mM cysteine during cooling and equilibrium processes(D-F). Different lower-case letters denote significant differences (p < 0.05). fresh: freshly ejaculated spermatozoa. cooled: freshly ejaculated spermatozoa cooled from room temperature to 5 ^o^C in the absence of cysteine. cooled-cys: freshly ejaculated spermatozoa cooled from room temperature to 5 ^o^C in the presence of cysteine. equil.: freshly ejaculated spermatozoa cooled from room temperature to 5 ^o^C and then equilibrated for 30 min at 5 ^o^C in the absence of cysteine. equil.-cys: freshly ejaculated spermatozoa cooled from room temperature to 5 ^o^C in the absence of cysteine and then equilibrated for 30 min at 5 ^o^C in the presence of cysteine.

A preliminary experiment was designed to detect whether addition of cysteine to freezing extender improves the quality of frozen-thawed rabbit spermatozoa via examination of sperm motility, plasma membrane integrity and intact acrosomes. Various concentrations of cysteine (0, 2.5, 5, 7.5, 10 mM) were added to the freezing extender. It was found that supplementation of 7.5 mM cysteine improved post-thaw motility, acrosome intactness, membrane integrity (p < 0.05; Fig 2A–2C), moreover, we also found that addition of cysteine significantly increased the percentage of acrosome-intact viable sperm (S1 Fig), and addition of 7.5 mM cysteine showed the highest value (33.0±1.6%), compared to the control (19.7±0.6%) (p<0.05, S1 Fig). Therefore, addition of 7.5 mM cysteine was used in the subsequent experiments to the TCG semen extender, freezing extender and, thawing solution.

First, sperm GSH content and activity of glutathione peroxidase were measured during the process of preservation and post-thaw incubation. Secondly, intracellular sperm ROS and H_2_O_2_ was assayed during these processes. Thirdly, to elucidate whether cysteine could improve sperm tolerance at each step of the process, sperm motility, acrosome intactness and membrane integrity were measured during cooling, equilibration, freeze-thaw and post-thaw incubation. At last, sperm-zona pellucida binding capacity, as sperm functional competence, was also examined.

## Results

### Glutathione (GSH)

**Fig 3:**
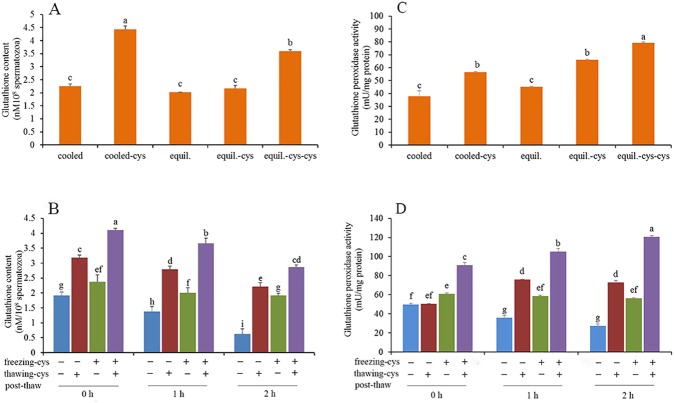
Effect of cysteine on glutathione content (A and B) and glutathione peroxidase activity in spermatozoa (C and D) during cooling, equlibrium, freezing-thawing and post-thaw incubation. Bars represent the mean ± SEM (n = 3). cooled: freshly ejaculated spermatozoa cooled from room temperature to 5^°^C in the absence of cysteine. cooled-cys: freshly ejaculated spermatozoa cooled from room temperature to 5^°^C in the presence of cysteine. equil.: freshly ejaculated spermatozoa cooled from room temperature to 5^°^C and then equilibrated for 30 min at 5^°^C in the absence of cysteine. equil.-cys: freshly ejaculated spermatozoa cooled from room temperature to 5^°^C in the absence of cysteine and then equilibrated for 30 min at 5^°^C in the presence of cysteine. equil.-cys-cys: freshly ejaculated spermatozoa cooled from room temperature to 5^°^C and then equilibrated for 30 min at 5^°^C. Spermatozoa were exposed to cysteine during both cooling and equilibrium. freezing-cys: Cysteine was added to freezing extender (+); cysteine was absent from the freezing extender (—). thawing-cys: Cysteine was added to thawing solution (+); cysteine was absent from the thawing solution (—).

As shown in Fig 3A, during the process of cooling from room temperature to 5°C, the addition of cysteine to the TCG extender increased GSH reducing activity, compared with the control (p < 0.05). Similarly, supplementation of both TCG extender and freezing extender with cysteine enhanced the GSH reducing activity as well (p < 0.05).

The GSH content of post-thaw spermatozoa during incubation at 37°C for 2 h is shown in Fig 3B. Compared with the control, at 0 h of incubation, addition of cysteine to either freezing extender or thawing solution led to an increase in GSH synthesis (p < 0.05). An increase in reducing activity was observed when cysteine was added to both the freezing extender and thawing solution (p < 0.05), which was also the case at 1 h and 2 h of incubation. These data indicate that addition of cysteine to the extenders resulted in more production of GSH during all the processes.

### Glutathione peroxidase activity

As shown in Fig 3C, exposure of spermatozoa to cysteine enhanced glutathione peroxidase activity during cooling and equilibration (p < 0.05). Exposure spermatozoa to cysteine during cooling and equilibration further increased the activity of glutathione peroxidase (p < 0.05). When compared with the control, addition of cysteine to the thawing solution led to higher activity of glutathione peroxidase at 1 h and 2 h of post-thaw incubation (p < 0.05, Fig 3D) although there was no significant difference for glutathione peroxidase activity at 0 h. Addition of cysteine to the freezing extender enhanced the activity of glutathione peroxidase at 0, 1 and 2 h (p < 0.05). In addition, addition of cysteine to the thawing solution resulted in higher values of glutathione peroxidase activity than that of adding cysteine to the freezing extender at 1 h and 2 h post-thaw incubation (p < 0.05). A synergistic augmentation was displayed when cysteine was added to both the freezing extender and thawing solution. Interestingly, the value of glutathione peroxidase activity increased during post-thaw incubation (p < 0.05). The data suggest that exposure of spermatozoa to cysteine enhances the activity of glutathione peroxidase during freeze-thaw and post-thaw incubation and the addition of cysteine to the both freezing extender and thawing solution maintains high activity of glutathione peroxidase in the post-thaw spermatozoa.

### Reactive oxygen species (ROS)

**Fig 4:**
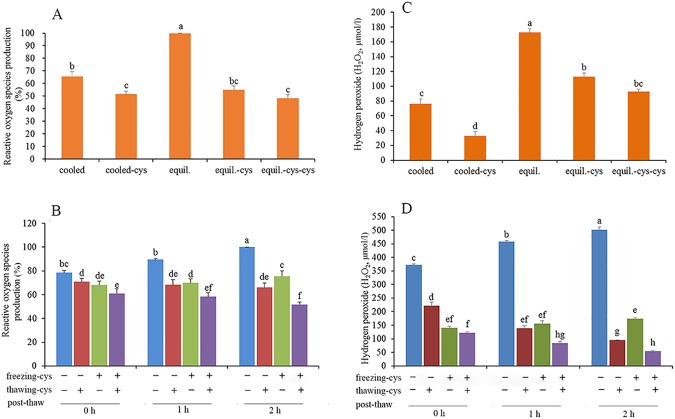
Effect of cysteine on reactive oxygen species production (A and B) and hydrogen peroxide (C and D) in spermatozoa during during cooling, equlibrium, freezing-thawing and post-thaw incubation. Bars represent the mean ± SEM (n = 3). cooled: freshly ejaculated spermatozoa cooled from room temperature to 5^°^C in the absence of cysteine. cooled-cys: freshly ejaculated spermatozoa cooled from room temperature to 5^°^C in the presence of cysteine. equil.: freshly ejaculated spermatozoa cooled from room temperature to 5^°^C and then equilibrated for 30 min at 5^°^C in the absence of cysteine. equil.-cys: freshly ejaculated spermatozoa cooled from room temperature to 5^°^C in the absence of cysteine and then equilibrated for 30 min at 5^°^C in the presence of cysteine. equil.-cys-cys: freshly ejaculated spermatozoa cooled from room temperature to 5^°^C and then equilibrated for 30 min at 5^°^C. Spermatozoa were exposed to cysteine during both cooling and equilibrium. freezing-cys: Cysteine was added to freezing extender (+); cysteine was absent from the freezing extender (—). thawing-cys: Cysteine was added to thawing solution (+); cysteine was absent from the thawing solution (—).

Addition of cysteine to the TCG extender or the freezing extender scavenged ROS (Fig 4A). As shown in Fig 4B, compared with the control, addition of cysteine to either freezing extender or thawing solution led to lower ROS values at 0, 1 and 2 h post-thaw incubation (p < 0.05). Similar results were observed between addition of cysteine to the freezing extender and to the thawing solution at 0 and 1 h after thawing. Supplementing both the freezing extender and thawing solution led to an added effect (p < 0.05).

### Hydrogen peroxide (H_2_O_2_)

The H_2_O_2_ level in spermatozoa accumulated during cooling, and dramatically increased further during equilibrium (Fig 4C). Addition of cysteine to either the TCG extender or freezing extender decreased H_2_O_2_ levels during cooling and the equilibration process, respectively (p< 0.05). As shown in Fig 4D, H_2_O_2_ was accumulated in spermatozoa during post-thaw incubation in the control (p < 0.05). Addition of cysteine to either the freezing extender or thawing solution resulted in a reduction of H_2_O_2_ values at 0, 1 and 2 h post-thaw incubation (p < 0.05). Moreover, addition of cysteine to both the freezing extender and thawing solution led to a further decrease in H_2_O_2_ levels (p < 0.05). The value of H_2_O_2_ displayed a tendency to decrease with time during post-thaw incubation.

### Sperm parameters

**Fig 5:**
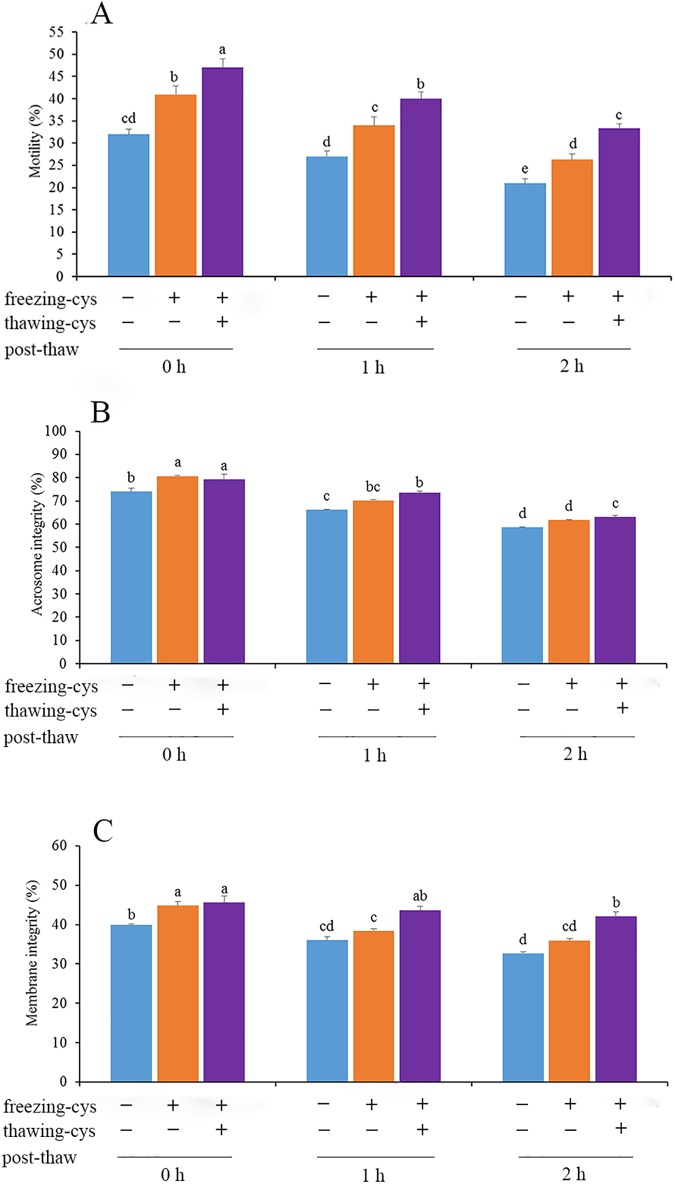
Effect of exposure spermatozoa to cysteine on motility, acrosome integrity and membrane integrity during post-thaw incubation. Bars represent the mean ± SEM (n = 5 independent replicates). Different lower-case letters denote significant differences (p < 0.05). freezing-cys: Cysteine was added to freezing extender (+); cysteine was absent from the freezing extender (—). thawing-cys: Cysteine was added to thawing solution (+); cysteine was absent from the thawing solution (—).

Addition of cysteine to TCG extender enhanced sperm motility, integrity of the acrosomal and plasma membranes during cooling (p < 0.05), compared with the control (Fig 2D–2F). Supplementation of the freezing extender with cysteine increased membrane integrity during cooling (p < 0.05, Fig 2F), and improved sperm motility at 0, 1 and 2 h post-thaw incubation. Addition of cysteine to both the freezing extender and thawing solution revealed a synergistic effect on sperm motility (p < 0.05, Fig 5A).

**Fig 6:**
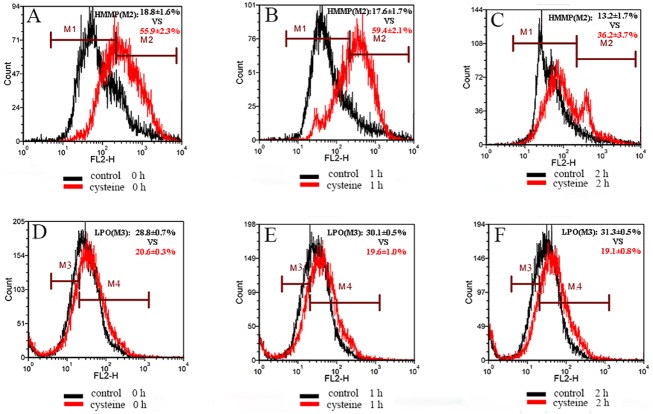
Effect of addition of L-cysteine to extender on sperm mitochondrial membrane potential (A,B,C) and lipid peroxidation (D,E,F) during incubation at 37°C for 2 h. Mitochondrial membrane potential was detected using JC-1 (lipophilic cation 5,5’,6,6,- tetrachloro-1,1’,3,3’, -tetraethylbenzimidazolcarbocyanine iodide) Mitochondrial Membrane Potential Detection Kit. Lipid peroxidation was measured by staining with probe BODIPY 581/591C11.Histogram shows spermatozoa with high (HMMP, marker M2) and low (marker M1) mitochondrial membrane potential in (A, B, C). Histogram shows spermatozoa with lipid peroxidation (LPO, marker M3) and without lipid peroxidation (marker M4) in (D, E, F). control: Spermatozoa that were frozen-thawed and incubated in absence of cysteine were used as the control. cysteine: Spermatozoa were frozen, thawed and incubated in the presence of cysteine.

For acrosomal intactness and membrane integrity, supplementation with cysteine increased the values at 0 h post-thaw incubation. If cysteine was added to the thawing solution, the values of acrosomal intactness and membrane integrity were enhanced at 1 and 2 h post thaw incubation (p < 0.05, Fig 5B and 5C), compared with the control. Moreover, at 0, 1 and 2 h of incubation, the percentages of spermatozoa with high mitochondrial membrane potential (red (yellow) fluorescence) in the cysteine group were 55.9 ± 2.3, 59.4 ± 2.1 and 36.2 ± 3.7, respectively, while those in the control group were 18.8 ± 1.6, 17.6 ± 1.7, 13.2 ± 1.7, respectively (Fig 6A–6C).

**Fig 7:**
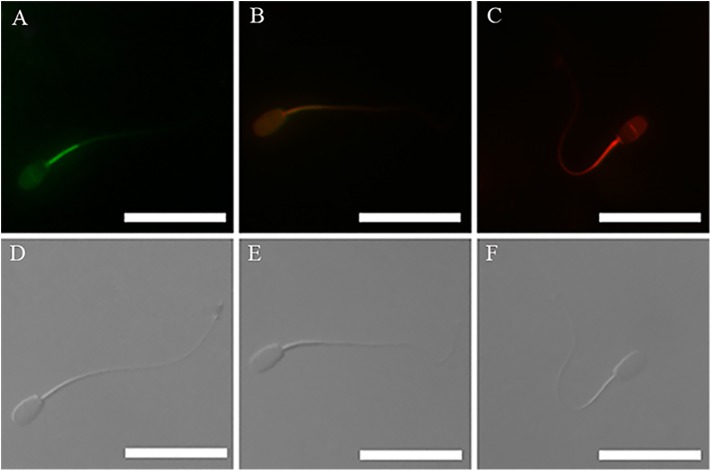
Photomicrographs of the post-thaw rabbit spermatozoa. (D, E, F) Images obtained under phase contrast microscope. images showed three kinds of spermatozoa stained with probe BODIPY 581/591C11 under the fluorescence microscope. (A) Image indicates a spermatozoon was serious oxidized; (B) Image indicates a spermatozoon was partially oxidized; (C) Image indicates a spermatozoon was not oxidized. Scale bars represent 30 μm.

In terms of lipid peroxidation, three kinds of spermatozoa were observed under the fluorescent microscope (Fig 7A–7C). The staining indicates that LPO occurred extensively in the head and mid-piece of the spermatozoa. On the contrary to the mitochondrial membrane potential, LPO values in the cysteine group showed a tendency to decrease (20.6 ± 0.3%, 19.6 ± 1.0%, 19.1 ± 0.8% for 0, 1 and 2 h) compared with the control group (28.8 ± 0.7%, 30.1 ± 0.5%, 31.3 ± 0.5% for 0, 1 and 2 h) (Fig 6D–6F) during 2 h of incubation.

**Fig 8:**
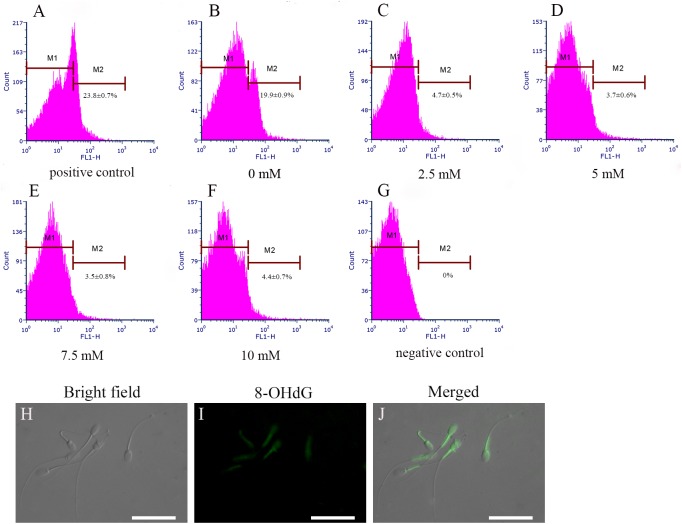
Effects of different concentrations of cysteine on 8-OHdG level of post-thaw rabbit spermatozoa (B-F). (A) positive control; (G) negative control. M1 indicates spermatozoa without 8-OHdG staining (8-OHdG-negative); M2 indicates spermatozoa with 8-OHdG staining (8-OHdG-positive). (H–J), 8-OHdG staining of the frozen-thaw spermatozoa: bright field (H); spermatozoa stained with propidium iodide (I); and merged image (J). Scale bars = 30 μm.

Sperm 8-hydroxydeoxyguanosine (8-OHdG) is a biomarker of sperm DNA oxidative damage. Addition of cysteine significantly decreased 8-OHdG level in the frozen-thawed spermatozoa. The percentage of 8-OHdG-positive spermatozoa significantly decreased with the supplementation of cysteine (p < 0.05, Fig 8B–8F). The percentage of spermatozoa with 8-OHdG fluorescence was 3.5 ± 0.8% in the 7.5 mM cysteine treatment and 19.9 ± 0.9% in the treatment without cysteine (p < 0.05, Fig 8), while it was 23.8± 0.7% in the positive control treated with 2 mM H_2_O_2_ and 1 mM FeCl_2_▪4H_2_O. Furthermore, as shown in Figs H-J, the distribution of 8-OHdG was in the midpiece and in the head of spermatozoa, indicating that both genomic DNA and mitochondrial DNA are altered by oxidative attack during cryopreservation.

### Sperm-zona pellucida binding capacity

**Fig 9:**
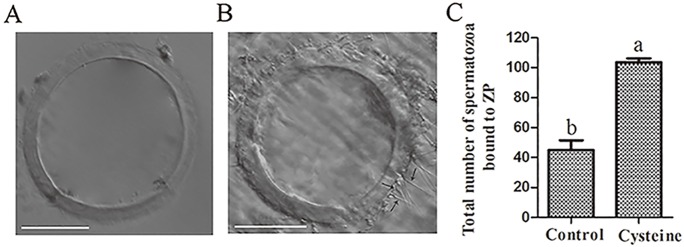
Effect of cysteine on sperm-zona pellucida (ZP) binding capacity of post-thaw spermatozoa in control and cysteine treatment. (A) zona pellucida, (B) sperm-zona pellucida complexes, black narrows indicate the sperm tightly bound to zona pellucida, (C) total nuber of sperm bound to zona pellucida. Bars represent the mean ± SEM (n = 3 independent replicates, each replicate was performed 25 zona pellucidas). Scale bars represent 100 μm.

The effect of cysteine on sperm-zona pellucida binding capacity was presented in Fig 9C. Addition of cysteine significantly increased the number of ZP-bound spermatozoa, compared to the control (103.7±6.5 vs 45.0±3.7, p < 0.05). The data indicated that addition of cysteine to the freezing extender was beneficial for spermatozoa to maintain their functional competence.

## Discussion

This study, for the first time, provides evidence that exposure of rabbit spermatozoa to cysteine enhances glutathione content and activated glutathione peroxidase activity during cryopreservation and post-thaw incubation. The present study showed that the addition of cysteine to the extenders significantly improved post-thaw motility, acrosome intactness, membrane integrity and mitochondrial potentials, and that this protective role was related to the prevention of ROS accumulation.

As a key antioxidant, glutathione protects cellular components from ROS attack [31]. The previous studies showed that the freeze-thaw process reduces the content of sperm glutathione in of boars [32], bulls [33] and goats [17]. And addition of glutathione to the freezing extenders improved the sperm quality [32, 34] though Marco-Jiménez et al. (2006) [35] reported that GSH had no effect on rabbit sperm during cooling and freezing process. In this study, we further observed that the sperm glutathione decreased at each step, including cooling, equilibrium, freeze-thaw and post-thaw incubation. Interestingly, the glutathione content increased when spermatozoa were exposed to cysteine during these processes, suggesting that cysteine enhances the ability of spermatozoa to synthesize glutathione during oxidation stress. Hanigan et al. (2014) [36] reported that cysteine was rate-limiting for glutathione synthesis in cells under oxidative stress. Cysteine was transported into cells via alanine-serine-cysteine [36] and cysteine/glutamate system [37]. It would be interesting to illuminate whether cysteine is incorporated into sperm cells. However, although the sperm samples were washed three times before analyzing of GSH content, the possibility cannot been excluded that the measurement of GSH content may be disturbed by cysteine that permeated inside of spermatozoa but did not take part in synthesis of GSH. It needs to further confirm the metabolism of cysteine in future study using radiolabeled means.

Interestingly, addition of cysteine to freezing extender resulted in increase of glutathione peroxidase activity, which can catalyze the toxic hydroperoxides [38] in the frozen-thawed goat [17] and bovine [11] spermatozoa. In the present study, addition of cysteine to the extenders improved the glutathione peroxidase activity at all the steps during cryopreservation. Importantly, glutathione peroxidase activity increased with the time during post-thaw incubation as well. Hamilton et al. (2016) [39] demonstrated that the activity of glutathione peroxidase was activated in case of necessity, especially in animals showing higher susceptibility to oxidative stress. During cryopreservation and post-thaw incubation, addition of cysteine resulted in increase of the glutathione content, which acted as a substrate for glutathione peroxidase and therefore the increased with glutathione would lead to activate the activity of glutathione peroxidase. Collectively, cysteine, which has antioxidant capacity, enhances glutathione content and activates glutathione peroxidase as well, thus the scavenging ROS. Moreover, it has been known that oxidized glutathione is transformed to its active form by NADPH-dependent function [40]. It is not clear that how NADPH regulates this reaction in spermatozoa and whether NADPH increases during the process of cryopreservation.

Mammalian sperm plasma membranes contain an extraordinarily high concentration of polyunsaturated lipids, making them extremely susceptible to free radicals, which leads to lipid peroxidation (LPO) [4]. LPO results in loss of membrane integrity and fluidity increased permeability [41]. This can lead to impaired motility, and abnormal morphology.

Peroxides are the most pernicious of the metabolic free radicals among which H_2_O_2_ is formed in highest quantities. H_2_O_2_ can move easily through different compartments and attack molecules within the cells. It has been shown that H_2_O_2_ is the primary ROS responsible for the human sperm damage [42]. As described in our previous study [23], ROS was major generated in sperm mitochondria, and in the literature, elevated ROS level has been reported during cooling, freezing and thawing in human [3], bovine [43] and ram [44] spermatozoa. ROS was produced during post-thaw incubation in bovine [45], ram [46] and red deer [47] spermatozoa. In the present study, we observed that rabbit sperm accumulated ROS and H_2_O_2_ during cooling, equilibration, freeze-thaw, and post-thaw incubation, which was in agreement with the aforementioned observation in the other animal spermatozoa. Importantly, addition of cysteine to TCG extenders/freezing extender/thawing solution scavenged ROS and H_2_O_2_ during these steps. Taken together, cysteine may enhance the synthesis of glutathione which is an antioxidant, and also stimulate the activity of glutathione peroxidase. Therefore, addition of cysteine protects spermatozoa from ROS attack during preservation and post-thaw incubation.

The motility, membrane integrity, acrosome intactness and mitochondrial potentials, DNA integrity and sperm-zona pellucida binding capacity are important parameters for evaluation of sperm quality and function. In the present study, we found that exposure rabbit spermatozoa to cysteine not only improved the above four parameters of frozen-thawed spermatozoa, but also enhanced the parameters during post-thaw incubation and increased motility and integrity of acrosome and plasma membrane during cooling process. These data indicate that cysteine protects rabbit spermatozoa from damage during cooling, freeze-thawing, and post-thaw incubation. In the previous studies, addition of cysteine to freezing extender increased the motility and membrane integrity of the frozen-thawed spermatozoa in cats [20], buffalos [14], goats [18, 19], rams [21], and boars [9]. Furthermore, addition of cysteine significantly decreased the level of 8-OHdG, which is a biomarker for DNA oxidative damage. The results were consistent with a decrease in ROS and H_2_O_2_ level supplementation of cysteine. The distribution of 8-OHdG fluorescence was in the midpiece and in the head of spermatozoa, which was similar to the results of Gonzalez-Rojo et al. [48] and Huang et al. [49], indicating that both genomic DNA and mitochondrial DNA are altered by oxidative attack, and that addition of cysteine would protect sperm from ROS attack on DNA during cryopreservation. Finally, supplementation of cysteine improved the percentage of the cryopreserved spermatozoa with intact acrosome in buffalos [12, 13], bulls [10] and boars [9]. In the present study, we also found that supplementation of cysteine increased the value of acrosome-intact viable in post-thaw sperm. However, a few reports showed that addition of cysteine to the cryopreserved medium did not improve the acrosome intactness [11, 15, 22]. These contradictory findings may be due to the extenders and antioxidative capacity in the extender and in spermatozoa.

In conclusion, addition of cysteine enhanced antioxidant GSH content and the activity of glutathione peroxidase, while lowered ROS and LPO level, which makes spermatozoa avoid ROS to attack DNA, the plasma membrane and mitochondrial. Therefore, cysteine protects spermatozoa against ROS-induced damages during cryopreservation and post-thaw incubation.

## Supporting information

S1 Fig Effect of cysteine on acrosome status of living sperm cells. FITC-PNA^-^/PI^-^: viable spermatozoa with intact acrosome.
(PDF)
