# Supplementary material for: Cysteine protects rabbit spermatozoa against reactive oxygen species-induced damages
Source: PLoS One. 2017 Jul 10;12(7):e0181110. doi: 10.1371/journal.pone.0181110 (PMC5507327; doi:10.1371/journal.pone.0181110)
Supplement: S1 Fig — FITC-PNA-/PI-: viable spermatozoa with intact acrosome. (PDF) [file pone.0181110.s001.pdf]

Assessment of acrosome status on living sperm cells

The acrosome status on living sperm cells was assessed using FITC-PNA/PI double staining. Briefly, spermatozoa were incubated with FITC-PNA solution (final concentration: 2.0 μg/mL) and Propidium iodide (PI) solution (final concentration:10 μM) for 10 min at 37 ℃. Subsequently, the samples were washed twice by centrifugation for 3 min at 800×g to remove the unbound probe, and analyzed by a flow cytometry with FL2 (535 nm excitation and 617 nm emission) for PI fluorescence and FL1 (488 nm excitation and 525 nm emission) for FITC-PNA fluorescence. Sperm were identified and placed in one of the four following populations: (1) viable spermatozoa with intact acrosomes (FITC-PNA$^-$/PI$^-$); (2) viable spermatozoa with damaged acrosomes (FITC-PNA$^+$/PI$^-$); (3) non-viable spermatozoa with damaged acrosomes (FITC-PNA$^+$/PI$^+$); (4) non-viable spermatozoa with intact acrosomes (FITC-PNA$^-$/PI$^+$). As showed in supplementary Fig 1, addition of cysteine significantly improved the percentage of viable spermatozoa with intact acrosome. Supplementation of 7.5 mM cysteine showed the highest value (33.0±1.6%), compared to the control (19.7±0.6%) ($p < 0.05$).

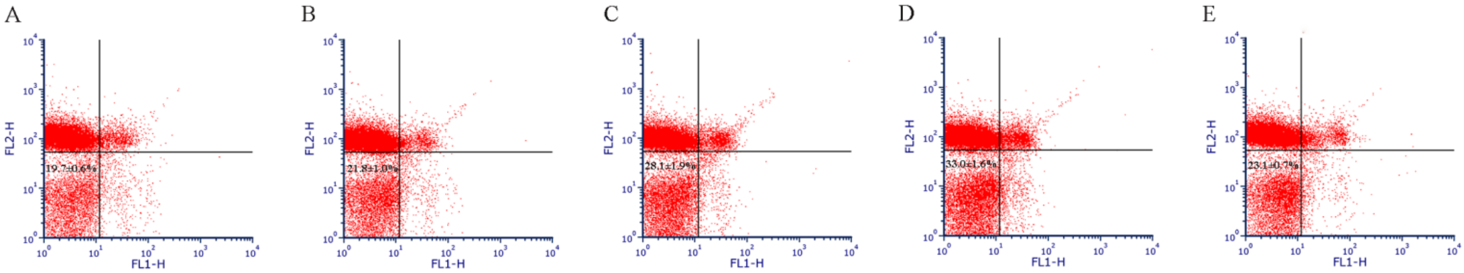

Supplementary Fig 1. Effect of cysteine on acrosome status of living sperm cells. FITC-PNA^{-}/PI^{-} : viable spermatozoa with intact acrosome.
